# Arterial thrombosis triggered by methotrexate-induced hyperhomocysteinemia in a systemic lupus erythematosus patient with antiphospholipid antibodies

**DOI:** 10.1186/s12959-023-00557-5

**Published:** 2023-11-03

**Authors:** Chiara Schiavi, Luca Marri, Simone Negrini

**Affiliations:** 1 Internal Medicine, Clinical Immunology and Translational Medicine Unit, IRCCS Ospedale Policlinico San Martino, Genoa, Italy; 2 Department of Internal Medicine, University of Genoa, Genoa, Italy

**Keywords:** Antiphospholipid antibodies, Methotrexate, Hyperhomocysteinemia, Systemic lupus erythematosus, Thrombosis

## Abstract

Systemic lupus erythematosus (SLE) patients have an increased risk of cardiovascular disease and thrombotic events, and the presence of antiphospholipid antibodies further raises the risk of these complications. Here we report a case of a patient with SLE and triple positivity for antiphospholipid antibodies who developed a popliteal artery thrombosis in the context of a severe hyperhomocysteinemia after the introduction of methotrexate (MTX) treatment. MTX is one of the most prescribed medications for a wide spectrum of autoimmune diseases, including SLE. On the other hand, by interfering with folate metabolism, it may induce hyperhomocysteinemia, which, in turn, may increase the risk of vascular complications. Current recommendations suggest screening and, when possible, treating classical and disease-related cardiovascular risk factors in all lupus patients. Based on what observed in our case, we suggest a follow-up of homocysteine levels after the introduction of drugs capable of inducing hyperhomocysteinemia, such as MTX, in SLE patients at high cardiovascular risk.

## Background

Antiphospholipid antibody syndrome (APS) is an acquired thrombophilia characterized by vascular thrombosis and/or obstetric complications in the presence of antiphospholipid antibodies (aPL). APS can occur as a primary condition or associated with another autoimmune disease, most commonly systemic lupus erythematosus (SLE). aPL can be detected in up to 50% of SLE patients, but only one third of these aPL carriers will eventually develop clinical manifestations [1]. In SLE patients, concomitance of a secondary APS, besides leading to an increased risk of thrombotic events, also increases cardiovascular disease (CVD) risk [2–4]. SLE is associated with a higher incidence of CVD as compared to the general population and this phenomenon is attributed to the interplay between traditional and disease-related CVD risk factors. Traditional risk factors include hypertension, dyslipidemia, smoking, diabetes, obesity, and family history of early CVD. SLE-related risk factors include disease duration, lupus activity, renal involvement and use of specific medications (e.g., glucocorticoids) [2, 4, 5]. Moreover, CVD risk factors as well as hereditary and acquired coagulation disorders increase the risk of thrombotic events in aPL positive patients [1, 2, 6].

It is widely accepted that the treatment goal for SLE should aim at disease activity control and prevention of flares employing the lowest possible dose of glucocorticoids (GCs) [5]. To this purpose, the use of immunosuppressive (IS) agents can facilitate the reduction, or even the discontinuation, of GCs. Among IS treatments commonly employed in SLE, low-dose methotrexate (MTX) (10–25 mg/weekly) is recommended for patients with articular or mucocutaneous manifestations with a mild to moderate disease activity [5]. MTX is an inhibitor of dihydrofolate reductase that decreases intracellular folates thus reducing the de novo synthesis of purines and pyrimidines crucial for cellular proliferation. Beside folate antagonism, it has been described a number of pleiotropic pharmacological effects on various immune cells leading to a dampening of the inflammatory response [7, 8].

Notably, the main reason leading to MTX withdrawal is related to adverse effects rather than lack of efficacy. The adverse effects associated with MTX are mainly attributed to folate antagonism and include nausea, mucositis, alopecia, cytopenia, interstitial lung disease and hepatotoxicity (most commonly liver enzyme elevation) [7]. In addition, interfering with folate metabolism, MTX may lead to hyperhomocysteinemia, thus increasing the risk of developing CVD and thrombotic events [9–11]. In order to reduce MTX toxicity, without significantly compromising its activity, folic acid supplementation is widely recommended in patients receiving MTX (e.g., 5 mg folic acid taken the day after the weekly MTX dose) [5, 7].

## Case presentation

A 56 years old Caucasian woman presented to the emergency department complaining intermittent claudication at left lower limb over last 3 weeks (Fontaine classification stage IIb).

Her past medical history included SLE (according to the 2019 EULAR/ACR SLE classification criteria [12]) with triple positive aPL antibody (high-risk) profile. Previous SLE manifestations included seizures, proliferative glomerulonephritis, Jaccoud's arthropathy and mitral regurgitation in Libman–Sacks endocarditis treated with mechanical valve replacement five years earlier. Her aPL profile was characterized by persistent presence of lupus anticoagulant (LA) along with high IgG titres of anti-cardiolipin (aCL) and anti-β2‐glycoprotein I (aβ2GPI) antibodies without previous pregnancy complications or history of thrombosis.

Patient’s home therapy included hydroxychloroquine, prednisone, pantoprazole, bisoprolol, cholecalciferol and warfarin with a target international normalized ratio (INR) of 2.5–3.5 as thromboprophylaxis following prosthetic valve replacement. Of note, INR was regularly monitored to maintain adequate prophylactic levels and previous INR values were repeatedly within the target range. Three months earlier, low-dose MTX (15 mg/weekly) was added to patient’s therapy due to recalcitrant arthritis and inability to reduce GCs daily dose to acceptable levels.

On physical examination, the left lower extremity was cold without trophic lesions or sensory-motor deficit. Both dorsalis pedis and posterior tibial pulses were absent on the left side.

Doppler ultrasonography of lower extremities documented left popliteal artery thrombosis whereas echocardiography showed a functioning prosthetic mitral valve without evidence of regurgitation or thrombosis. The patient underwent percutaneous transluminal angioplasty without stent and low-dose acetylsalicylic acid was added to ongoing therapy.

Laboratory tests evidenced mild leukopenia (2800 WBC per mmc), haemoglobin 12.6 g/dL, platelet count 126,000 per mmc, normal hepatic tests and mild hypocomplementemia C3 83 mg/dl (n.v. 90–180) and C4 16 mg/dl (n.v. 20–50). INR was 3.1. Total proteinuria (< 150 mg/L) and renal function tests were normal. Immunological profile revealed ANA 1:320 (homogeneous pattern), high titre anti-dsDNA antibodies (> 1:80), aCL IgG > 280 (n.v. 0–20) and aβ2GPI IgG 96 (n.v. 0–20). aPL antibodies were assessed by chemiluminescent immunoassay (CLIA).

Besides aPL, patient did not present any evident acquired thrombophilia factor such as smoking, trauma, oestrogen-based treatments, immobilization, obesity, nephrotic syndrome, or cancer. Factor V Leiden and prothrombin G20210A mutation were anamnestically negative whereas protein C, protein S and antithrombin III levels, normal in previous assessments, and LA were not retested because their low reliability during an active clotting event and in patients on anticoagulation therapy. However, serum homocysteine levels were abnormally high at 68.1 mcg/L (n.v. 5–15 mcg/L) in the absence of methylenetetrahydrofolate reductase (MTHFR) mutations. Therefore, B-vitamin complex supplementation was introduced, while MTX was discontinued. Two months upon the initiation of this therapy, homocysteine levels were normalized to 8.7 mcg/L. On the basis of current classification criteria, the patient was finally classified as secondary APS associated with SLE [13].

## Discussion and conclusions

Patients with SLE have an increased CVD risk conferred by disease-specific and traditional risk factors and aPL antibodies, when present, represents another important CVD risk factor [2, 3, 14]. aPL-related CVD risk depends on inflammatory and atherothrombotic mechanisms including accelerated atherosclerosis, dysregulation of the coagulation cascade, endothelial dysfunction, cytokines and adhesion molecules induction, neutrophil extracellular traps release as well as activation of monocyte, neutrophils, platelet and complement pathway [2, 14].

The patient here described, despite her persistent high-risk aPL profile, never developed known or diagnosed arterial and/or venous thrombosis until this episode. Notably, at the time of the thrombotic event, patient was already on anticoagulation for valve replacement. Therefore, she was receiving a full anticoagulation regimen reserved for those APS patients having already experienced thrombotic manifestations (secondary thromboprophylaxis) [6].

It is unclear why high aPL titres may persist for years in asymptomatic patients and thrombotic events occur only occasionally. The currently accepted “two-hit hypothesis” proposes that aPL increase the risk of thrombotic events (“first hit”) but thrombus formation takes place only if another procoagulant condition intervenes (“second hit”) [1]. In our case, we suppose that the recently introduced MTX therapy may have increased homocysteine plasma levels, and hyperhomocysteinemia may have acted as a “second hit” in precipitating the thrombotic event observed. Accordingly, high levels of homocysteine are known to cause vascular injury through different mechanisms including, among others, disturbance of the balance between procoagulant and anticoagulant factors, increased production of reactive oxygen species, and impairment of nitric oxide synthesis [15, 16]. Our hypothesis would be further supported by the fact that homocysteine levels, tested before MTX introduction, were repeatedly normal, and no other additional prothrombotic factors were identified.

In the setting of SLE, hyperhomocysteinemia has been linked to an increased risk of atherosclerosis and thrombotic events [4, 17–22]. Therefore, in SLE patients with hyperhomocysteinemia, B vitamin supplementation is usually recommended and, although the efficacy of this approach is controversial [23], it is generally agreed that its potential benefits outweigh cost or safety concerns [4, 19, 22, 24].

Current recommendations underline the importance of CVD in SLE and APS, and recommend that all patients be screened for CVD risk factors (Table 1) [2, 5, 6]. On the basis of the case reported and on the evidence in the literature, we suggest considering homocysteine as part of routine screening in all SLE patients, and we recommend, especially for those patients with a high-risk CVD profile (e.g., triple aPL positive), monitoring homocysteine levels after the introduction of drugs that may induce hyperhomocysteinemia, such as MTX.

**Table 1:** Cardiovascular risk management in systemic lupus erythematosus and antiphospholipid syndrome [integrated and adapted from [2, 5, 6]]

1. SLE diagnosis
2. Check cardiovascular/thrombotic risk factors (at diagnosis and repeat according to individual patient characteristics and risk levels), including: - aPL antibodies (if present define the aPL-associated risk profile) - Traditional CVD risk factors (e.g., smoking, obesity, dyslipidemia, hypertension, diabetes, CVD family history) - SLE-specific CVD risk factors (disease duration, disease activity, renal disease, GCs, *hyperhomocysteinemia*) - Acquired hypercoagulability (e.g., major surgery/trauma, immobilization, pregnancy, malignancies, smoking, nephrotic syndrome) - Hereditary hypercoagulability (e.g., factor V Leiden, prothrombin mutation, protein C deficiency ^a^, protein S deficiency ^a^, antithrombin deficiency ^a^, elevated factor VIII activity ^a^, *hyperhomocysteinemia* ^a^)
3. Correct any modifiable risk factor (lifestyle modifications, pharmacologic therapies) and: - Hydroxychloroquine is recommended for all SLE patients (unless contraindicated) and should also be considered to reduce the risk of CVD - Low disease activity (at the lowest possible GCs dose) should be maintained to also reduce CVD risk - In asymptomatic aPL carriers (no history of thrombosis or pregnancy complications) prophylactic treatment with LDA is recommended, particularly in high-risk aPL profile patients - In patients with definite APS, long-term VKA is recommended (if recurrent thrombosis despite adequate VKA treatment, an increase of INR target or addition of LDA should be considered)

*aPL* Antiphospholipid, *APS* Antiphospholipid syndrome, *CVD* Cardiovascular disease, *GCs* Glucocorticoids, *INR* International normalised ratio, *LDA* Low-dose aspirin, *SLE* Systemic lupus erythematosus, *VKA* Vitamin K antagonists

^a^these factors can be hereditary or acquired

## Data Availability

Not applicable.

## Abbreviations

aCL: Anti-cardiolipin
aβ2GPI: Anti-β2-glycoprotein I
aPL: Antiphospholipid
APS: Antiphospholipid syndrome
CVD: Cardiovascular disease
GCs: Glucocorticoids
INR: International normalized ratio
LA: Lupus anticoagulant
LDA: Low-dose aspirin
MTHFR: Methylenetetrahydrofolate reductase
MTX: Methotrexate
SLE: Systemic lupus erythematosus
VKA: Vitamin K antagonists
